# Investigation of Conical Spinneret in Generating More Dense and Compact Electrospun Nanofibers

**DOI:** 10.3390/polym10010012

**Published:** 2017-12-22

**Authors:** Aya Hamed, Nader Shehata, Mohamed Elosairy

**Affiliations:** 1 Department of Engineering Mathematics and Physics, Faculty of Engineering, Alexandria University, Elhadara, Alexandria 21544, Egypt; aya_ali@mena.vt.edu (A.H.); melosairy@hotmail.com (M.E.); 2 Center of Smart Nanotechnology and Photonics (CSNP), Smart Critical Infrastructure (SmartCI) Research Center, Alexandria University, Elhadara, Alexandria 21544, Egypt; 3 USTAR Bio Innovations Center, Faculty of Science, Utah State University, Logan, UT 84341, USA; 4 Department of Physics, Kuwait College of Science and Technology (KCST), Doha Road, 7th Ring Road, P.O. Box 27235, Safat 13133, Kuwait

**Keywords:** electrospinning, nanofibers, conical spinneret, electric field distribution, discrete bead model

## Abstract

Electrospinning is an important, widely used process to generate nanofibers. However, there is still an open window for different designs of both spinneret and collector electrodes to be investigated. This paper introduces the impact of new design of conical spinneret electrode on the generated electrospun nanofibers. In this work, the conical feeder is used to generate electrospun Poly(vinyl alcohol) (PVA) nanofibers, and being compared to the traditional needle feeder at the same processing conditions. The jet’s mechanism is simulated using discrete bead model along with estimated calculations of both deposition area and fiber radius. The electric field distribution that is around the charged cone is analyzed. Based on both theoretical modeling and experimental measurements, a comparison of mean diameter, deposited area, and the thickness of generated nanofibers is presented related to both conical and needle electrodes. Conical feeder shows clearly compact nanofibers mat in terms of deposition area (spherical deposition of diameter ~6 cm) up to half-area of needle deposited nanofibers with high fiber density for the same time of the process. Moreover, the conical electrode is found to have privilege in terms of productivity rate and operation time. This study can be useful in generating localized nanofibers within different applications, such as biomedical tissue scaffolds, textile, and sensors.

## 1. Introduction

Electrospinning is an electrostatic drawing process for the fabrication of nanofibers. Electrospinning embraces a simple and versatile technique for the fabrication of fibers with diameters in the range of 100 nm or less. The formation of nanofibers through electrospinning is based on stretching and elongation of solution or melts due to electrostatic forces. This process allows for the drawing of solutions or melts into nanofibers. There are plenty of potential applications for electrospun nanofibers, including nano-catalysis, tissue scaffolds, protective clothing, filtration, and nano-electronics [1,2,3,4].

In electrospinning, polymer solution is forced through a syringe, and then a solution drop is formed at the tip of the needle. A high voltage is applied to the needle, which induces electric charges within the fluid. When the applied voltage exceeds a critical voltage, typically more than 5 kV, the repulsive force within the charged solution is larger than its surface tension and a jet erupts from the tip of the needle. Then, the jet is accelerated toward the electrically grounded collector. As this jet travels through the air, the solvent evaporates, and consequently a polymer fiber is collected on an electrically-grounded target [2,5,6].

Electrostatic interactions between individual charge elements in the jet and between charge elements are responsible for initiation of bending instability and splitting instabilities. When the electrostatic forces have become large enough, they cause the jet to attempt to force itself away, thus causing jet splitting along with the observation of both motions of bending and whipping. Therefore, electrostatic forces play a large role in the mechanics of the bending and splitting instabilities. As bending instability is initiated, it is sustained due to absence of any force to counter it; the small perturbations grow, leading to chaotic motion of jet. This chaotic motion of the electrospinning jet, as it travels to its target, causes random deposition of electrospun fibers on the collector [6]. The development of the bending instability enables such large cross-sectional reduction of the jet into the nano-scale [7].

Since electrospinning is caused by charges on the jet, these charges can be influenced by the external electric field that affects on the jet’s path. The electrospinning jet can be controlled through changing the electric field profile between both the source of the jet, or what we call spinneret, and the grounded collector [2]. It should be possible to dampen the bending instability through controlling both the distribution and strength of the electric field between the spinneret and collector [6].

There were some recent research works that studied the control of nanofibers deposition based on electric field manipulation. Jaeger et al. used a single ring electrode to stop the chaotic motion of the electrospinning jet [8]. Deitzel et al. used eight ring auxiliary electrodes to eliminate the whipping instability, thereby reducing the deposition area [6]. Kim et al. applied a cylindrical auxiliary electrode to multi-spinneret to stabilize the initial polymer jet from the nozzle, which resulted in size reduction of the deposited fiber area [9]. The superposition principle of electric fields as a method of controlled deposition in electrospinning was investigated in reference [10]. Wu et al. positioned three auxiliary rod electrodes behind a rotating collection mandrel to serve further focus of the electrostatic field and constrain the deposition [11]. Also, Arras et al. used auxiliary electrodes to control the fiber path and the deposition area of the electrospun fibers [12]. The auxiliary electrodes were used for the generation of a symmetrical auxiliary electric field that narrowed the electrospinning jet bending instability. However, Neubert et al. used simple electrostatic lens systems to control deposition area of the electrospun [13]. While Bellan et al. used both a direct current (DC) focusing field to reduce the characteristic spot size along with a time-varying jet-steering field. That constrained flight path by varying the potential difference [14]. That could cause the fiber flight path to alternative scan on the collector electrode. In another research work, Shafiei et al. used a two rings electrospinning setup that offered a control over the location and size of the deposition area down to a few millimeters [15].

This work presents a new study of a conical spinneret as a method to control the generated electric field in the electrospinning process. The conical spinneret provides a stronger electric field when compared to the traditional needle feeder. In this paper, a comparison between both conical and needle feeders is presented with the impact on the resulting radius and thickness of the formed nanofibers mat. In addition, a verification of the results has been theoretically analyzed via discrete bead model of Reneker [16].

## 2. Experimental Study

### 2.1. Electrospinning Process

**Figure 1:**
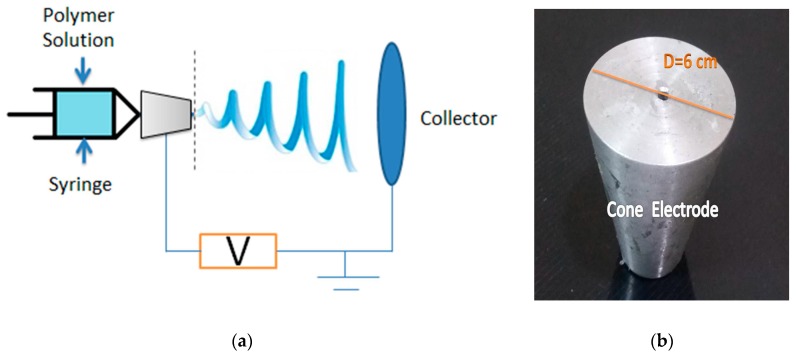
(**a**) Schematic of the electrospinning setup and the used cone feeder; and, (**b**) Cone spinneret electrode dimensions.

Typical electrospinning setup consists of a high voltage power supply ranges up to 30 kV, a syringe pump that is used to regulate the feed rate of polymer solution, a syringe to hold the polymer solution, and a collector plate that is covered with aluminum foil used as a target where nanofibers deposit, as shown in the Figure 1. The voltage power supply is connected to the cone or needle, while the collector is grounded.

The electrospinning setup in our lab consists of a high voltage power supply (Spellman High Voltage Electronics Corporation model CZE1000R, Hauppauge, New York, NY, USA), a syringe pump (NE1000-Single Syringe Pump, New Era, Farmingdale, New York, NY, USA), with a connected 5 mL plastic syringe of 18 gauge metallic needle and a circular metallic collector of radius 10 cm covered with aluminum foil is used as a target. The used conical feeder has been manufactured with top diameter of 30 mm, base diameter 60 mm, height 70 mm, and hole diameter 4 mm. A rubber tube (PTFE Teflon Tube 2 mm ID 4 mm OD For 1.75 mm, RepRap HM, Guangzhou, China) is used to deliver the solution from the syringe to the cone electrode.

### 2.2. Synthesis, Process Parameters, and Characterization of Nanofibers

Poly(vinyl alcohol) (PVA) of molecular weight (*M*_w_ = 61,000 g/mol) was purchased from Sigma-Aldrich (St. Louis, MO, USA). It was used without further purification. A concentration of 10 wt % PVA solution was prepared by mixing 10 g PVA pellets with 90 mL of distilled water. The solution was heated to 100 °C for 30 min, and then it was stirred overnight. The prepared sample of PVA is pumped to the syringe and potential difference of 15–25 kV range is applied between the needle and collector at a distance 15 cm and constant pumping rate 2 mL/h., causing the jet to initiate from the tip of a needle. The solvent evaporates leaving behind fibers to be collected randomly on the grounded target. The average operation time was 30 min. The effect of some parameters was determined experimentally. The resulting fibers’ diameters were measured and radius distributions were compared. The distance from the tip of needle to the collector was varied from 10 to 20 cm, while feed rate and voltage were constant. Feed rate was varied from 1 to 5 mL/h., and the resulting fibers’ diameter and deposition area were examined. Finally, the modifications to the electrospinning setup were tested for their validity and the resulting fiber samples were analyzed using SEM to study the characteristics of the produced fibers (radius, deposition area, and thickness of fiber mat). The electrospun fibers were observed with a scanning electron microscope SEM (JSM-5910LV, Peabody, MA, USA), and it is used the software of IMAGEJ (NIH Image, Bethesda, MD, USA) as an image analysis software to determine both the fiber mean diameters and mat thickness.

## 3. Analytical Study

Two major modeling zones have been identified in the electrospinning jet. These zones are: zone close to the capillary outlet where an axi-symmetric jet exits and thins down and the whipping instability zone where the jet spirals and accelerates towards the collector plate. Our interest is in the whipping instability zone where most of the thinning of the jet occurs. The whipping instability region is responsible for reducing fibers into the nano-scale and obtaining final deposition area. The discrete bead model of Reneker et al. has been used in this work to simulate the bending (whipping) instability zone and to study the effect of different parameters on the electrospinning process and the resulting nanofibers radius distribution and deposition area [16].

### 3.1. Discrete Bead Model

**Figure 2:**
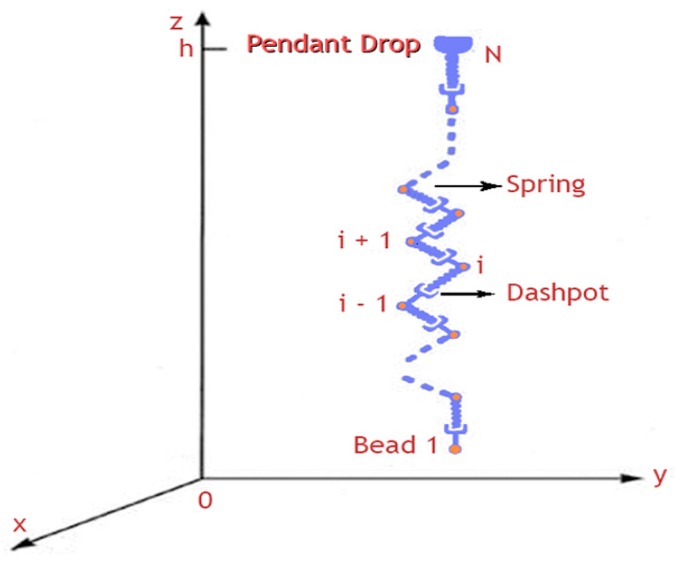
Bending electrospun jet modeled by a system of beads connected by visco-elastic elements [16].

To describe the jet flow path, the fluid jet is modeled as a system of beads that are connected by visco-elastic elements consists of a spring and a dashpot, as shown in Figure 2. The model assumes that the jet is split into several equal segments and each segment is modeled as visco-elastic dumbbell connecting two successive beads, where each bead has a charge e and mass m. The net force acting on each bead consists of visco-elastic force, Coulomb force, surface tension force, and an electric force due to the applied electric field [16].

For a given bead i, it is connected to two beads a preceding bead (i − 1) and a subsequent bead (i + 1). The visco-elastic stress between each two beads is given by

(1) $$\frac{{d\sigma }_{\mathrm{bi}}}{\mathrm{dt}}=\frac{G}{l_{\mathrm{bi}}}\frac{{\mathrm{dl}}_{\mathrm{bi}}}{\mathrm{dt}}-\frac{G}{\mu }\sigma_{\mathrm{bi}}$$

(2) $$\frac{{d\sigma }_{\mathrm{ui}}}{\mathrm{dt}}=\frac{G}{l_{\mathrm{ui}}}\frac{{\mathrm{dl}}_{\mathrm{ui}}}{\mathrm{dt}}-\frac{G}{\mu }\sigma_{\mathrm{ui}}$$

where $\sigma_{\mathrm{bi}}$ is the visco-elastic stress between bead i and the preceding bead, $\sigma_{\mathrm{ui}}$ is the visco-elastic stress between bead i and the subsequent bead, $l_{\mathrm{ui}}$ represents the filament length connecting bead i to subsequent bead, $l_{\mathrm{bi}}$ represents the filament length connecting bead i to the preceding bead, and G is the elastic modulus.

Through performing the momentum balance at each bead, the equation of jet path can be obtained. According to Newton’s 2nd law, the forces acting on ith bead are summed and equated to mass multiplied by acceleration. The terms in the right hand side of Equation (3) represents forces that are acting on bead number (i) which are the net Coulomb force acting on ith bead from all the other beads, force due to the electric field, visco-elastic force, and surface tension force.

(3) $$m\frac{d^{2}r_{i}}{{\mathrm{dt}}^{2}\;}=\overset{N}{\underset{\begin{array}{c} j=1\\ j\neq i \end{array}}{\sum }}\frac{e^{2}}{R_{\mathrm{ij}}^{2}}\frac{\left(r_{i}-r_{j}\right)}{R_{\mathrm{ij}}}-\frac{{\mathrm{eV}}_{0}}{h}\hat{k}+\pi (\frac{a_{\mathrm{ui}}^{2}\sigma_{\mathrm{ui}}}{l_{\mathrm{ui}}}\left(r_{i+1}-r_{i}\right)-\frac{a_{\mathrm{bi}}^{2}\sigma_{\mathrm{bi}}}{l_{\mathrm{bi}}}\left(r_{i}-r_{i-1}\right))\;-\frac{{\alpha \pi a}_{\mathrm{avg}}^{2}K_{i}}{{\left(x_{i}^{2}{+y}_{i}^{2}\right)}^{\frac{1}{2}}}(\hat{i}\left|x_{i}\right|\mathrm{sign}\left(x_{i}\right)+\hat{j}|y_{i}|\mathrm{sign}\left(y_{i}\right))$$

where x, y and z are the Cartesian coordinates of the beads, while $\hat{i}$, $\hat{j}$, and $\hat{k}$ are the unit vectors along x, y, and z axes, a is the radius of the jet. R_ij_ is the distance between two bead, and is given by

(4) $$R_{\mathrm{ij}}={\left({{(x}_{i}-x_{j})}^{2}{\;+\;(y}_{i}-y_{j}{)}^{2}{\;+\;(z}_{i}-z_{j}{)}^{2}\right)}^{\frac{1}{2}}$$

where V_0_ is the applied voltage and h is the distance between pendant drop and collector. The coefficient α represents the surface tension coefficient, and K_i_ is jet curvature, which can be obtained by considering the definition found in [17].

(5) $$K_{i}\;=\frac{1}{r_{0}}$$

where r_0_ is the radius of curvature of ith bead, which can be represented as follows

(6) $$r={\left[\begin{array}{l} {\left({(x}_{i}-\frac{\left(x_{i+1}^{2}-x_{i}^{2}{+y}_{i+1}^{2}-y_{i}^{2}\right){\cdot (y}_{i-1}-y_{i})-\left(x_{i-1}^{2}-x_{i}^{2}{+y}_{i-1}^{2}-y_{i}^{2}\right){\cdot \;(y}_{i+1}-y_{i})}{{2((x}_{i-1}-x_{i}{)\;(y}_{i+1}-y_{i}{)-(x}_{i+1}-x_{i}){\cdot \;(y}_{i-1}-y_{i}))}\right)}^{2}\\ +{\left(y_{i}-\frac{\left(x_{i+1}^{2}-x_{i}^{2}{+y}_{i+1}^{2}-y_{i}^{2}\right){\cdot (x}_{i-1}-x_{i})-\left(x_{i-1}^{2}-x_{i}^{2}{+y}_{i-1}^{2}-y_{i}^{2}\right){\cdot \;(x}_{i+1}-x_{i})}{{2((x}_{i-1}-x_{i}{)\;(y}_{i+1}-y_{i}{)-(x}_{i+1}-x_{i}){\cdot \;(y}_{i-1}-y_{i}))}\right)}^{2} \end{array}\right]}^{\frac{1}{2}}$$

where x_i_, x_i+1_, and x_i−1_ are the x-coordinates of bead (i) and the subsequent bead (i + 1) and the preceding bead (i − 1). y_i_, y_i+1_, and y_i−1_ are the y-coordinates of bead (i) and the subsequent bead (i + 1) and the preceding bead (i − 1).

Here, it is worth mentioned that the effects of air drag force and gravity are negligibly small and do not affect the development of jet path. Force due to gravity can be considered as a secondary effect and can be neglected. The air drag force, which tends to compress the jet along its axis is smaller in comparison to stretching of gravity, and much smaller than stretching due to electrical forces that tends to elongate the jet [7].

The term V_0_/h represents the uniform electric field value inside the electrospinning process. This term would be modified by the electric field distribution from the conical electrode, which will be shown later in Section 4.1.

To obtain the dimensionless form of Equations (1)–(3), we use the dimensionless parameters shown in Table 1. The time t is divided by the relaxation time μ/G, stress is divided by G, velocity is rationalized by LG/μ, and radius (a) is normalized by a_0_. Therefore, the final forms of the system’s equations are as follows:

(7) $$\frac{d{\bar{\sigma }}_{ui}}{d\bar{t}}=\frac{1}{{\bar{I}}_{ui}}\frac{{dl}_{ui}^{¯}}{d\bar{t}}-{\bar{\sigma }}_{ui}$$

(8) $$\frac{d{\bar{\sigma }}_{bi}}{d\bar{t}}=\frac{1}{{\bar{I}}_{bi}}\frac{{dl}_{bi}^{¯}}{d\bar{t}}-{\bar{\sigma }}_{bi}$$

(9) $$m\frac{d^{2}{\bar{r}}_{i}}{{d\bar{t}}^{2}\;}=\overset{N}{\underset{\begin{array}{c} j=1\\ j\neq i \end{array}}{\sum }}Q\frac{\left({\bar{r}}_{i}-{\bar{r}}_{j}\right)}{{\bar{R}}_{\mathrm{ij}}^{3}}-V\hat{k}+F_{\mathrm{ve}}{\bar{a_{\mathrm{ui}}}}^{2}{\bar{\sigma }}_{\mathrm{ui}}\frac{\left({\bar{r}}_{i+1}-{\bar{r}}_{i}\right)}{{\bar{l}}_{\mathrm{ui}}}-F_{\mathrm{ve}}{\bar{a_{\mathrm{bi}}}}^{2}{\bar{\sigma }}_{\mathrm{bi}}\frac{\left({\bar{r}}_{i}-{\bar{r}}_{i-1}\right)}{{\bar{l}}_{\mathrm{bi}}}\;-A{\bar{K}}_{i}\frac{{\bar{a}}_{\mathrm{avg}}^{2}}{\sqrt{\left({\bar{x}}_{i}^{2}+{\bar{y}}_{i}^{2}\right)}}(\hat{i}\left|x_{i}\right|\mathrm{sign}\left(x_{i}\right)+\hat{j}|y_{i}|\mathrm{sign}\left(y_{i}\right))$$

Equation (9) is split into three Equations (9a)–(9c), in the x, y, and z directions, respectively. The three equations, together with Equations (7) and (8), represent the system’s equations. The set of equations is numerically solved for every bead in the jet in order to study the jet dynamics, the jet path, and the evolution of the position of beads with time.

(9a) $$m\frac{d^{2}{\bar{x}}_{i}}{{d\bar{t}}^{2}\;}=\overset{N}{\underset{\begin{array}{c} j=1\\ j\neq i \end{array}}{\sum }}Q\frac{\left({\bar{x}}_{i}-{\bar{x}}_{j}\right)}{{\bar{R}}_{\mathrm{ij}}^{3}}+F_{\mathrm{ve}}{\bar{a_{\mathrm{ui}}}}^{2}{\bar{\sigma }}_{\mathrm{ui}}\frac{\left({\bar{x}}_{i+1}-{\bar{x}}_{i}\right)}{{\bar{l}}_{\mathrm{ui}}}-F_{\mathrm{ve}}{\bar{a_{\mathrm{bi}}}}^{2}{\bar{\sigma }}_{\mathrm{bi}}\frac{\left({\bar{x}}_{i}-{\bar{x}}_{i-1}\right)}{{\bar{l}}_{\mathrm{bi}}}-A{\bar{K}}_{i}\frac{{\bar{a}}_{\mathrm{avg}}^{2}}{\sqrt{\left({\bar{x}}_{i}^{2}+{\bar{y}}_{i}^{2}\right)}}\left|{\bar{x}}_{i}\right|\mathrm{sign}\left(x_{i}\right)$$

(9b) $$m\frac{d^{2}{\bar{y}}_{i}}{{d\bar{t}}^{2}\;}=\overset{N}{\underset{\begin{array}{c} j=1\\ j\neq i \end{array}}{\sum }}Q\frac{\left({\bar{y}}_{i}-{\bar{y}}_{j}\right)}{{\bar{R}}_{\mathrm{ij}}^{3}}+F_{\mathrm{ve}}{\bar{a_{\mathrm{ui}}}}^{2}{\bar{\sigma }}_{\mathrm{ui}}\frac{\left({\bar{y}}_{i+1}-{\bar{y}}_{i}\right)}{{\bar{l}}_{\mathrm{ui}}}-F_{\mathrm{ve}}{\bar{a_{\mathrm{bi}}}}^{2}{\bar{\sigma }}_{\mathrm{bi}}\frac{\left({\bar{y}}_{i}-{\bar{y}}_{i-1}\right)}{{\bar{l}}_{\mathrm{bi}}}-A{\bar{K}}_{i}\frac{{\bar{a}}_{\mathrm{avg}}^{2}}{\sqrt{\left({\bar{x}}_{i}^{2}+{\bar{y}}_{i}^{2}\right)}}\left|{\bar{y}}_{i}\right|\mathrm{sign}\left(y_{i}\right)$$

(9c) $$m\frac{d^{2}{\bar{z}}_{i}}{{d\bar{t}}^{2}\;}=\overset{N}{\underset{\begin{array}{c} j=1\\ j\neq i \end{array}}{\sum }}Q\frac{\left({\bar{z}}_{i}-{\bar{z}}_{j}\right)}{{\bar{R}}_{\mathrm{ij}}^{3}}-V\hat{k}+F_{\mathrm{ve}}{\bar{a_{\mathrm{ui}}}}^{2}{\bar{\sigma }}_{\mathrm{ui}}\frac{\left({\bar{z}}_{i+1}-{\bar{z}}_{i}\right)}{{\bar{l}}_{\mathrm{ui}}}-F_{\mathrm{ve}}{\bar{a_{\mathrm{bi}}}}^{2}{\bar{\sigma }}_{\mathrm{bi}}\frac{\left({\bar{z}}_{i}-{\bar{z}}_{i-1}\right)}{{\bar{l}}_{\mathrm{bi}}}$$

### 3.2. Finite Element Analysis for Electric Field Distribution

**Figure 3:**
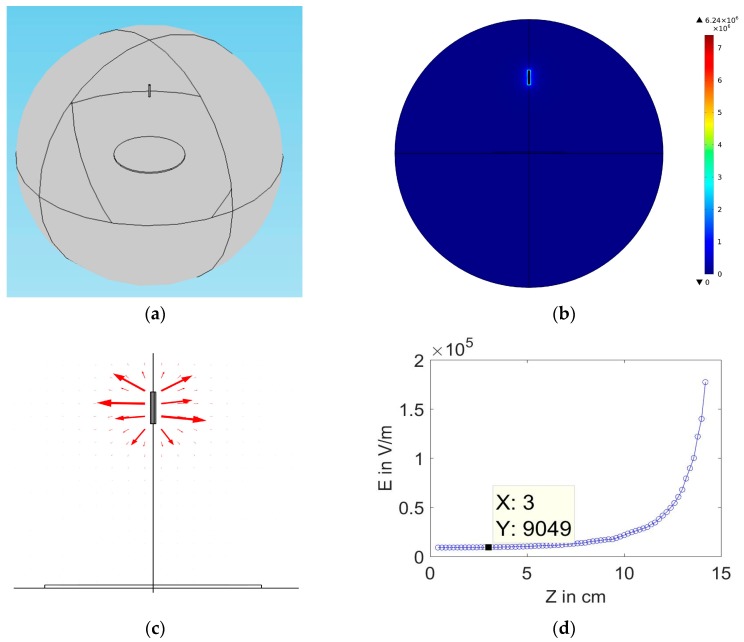
(**a**) Needle in typical electrosspinning setup in COMSOL software, (**b**) Electric field distribution surrounding to the needle spinnert at (d = 15 cm, V = 15 kV), (**c**) Mapping of electric field lines, and (**d**) Variation of electric field magnitude along z-axis, which presents the distance between the feeder and collector.

**Figure 4:**
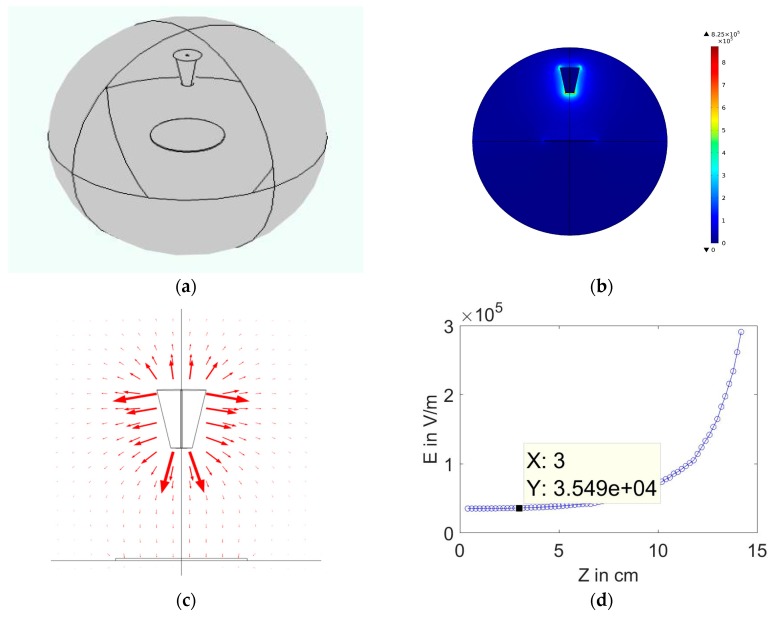
(**a**) Designed cone feeder in COMSOL software, (**b**) Electric field distribution surrounding to the conical spinnerte at (d = 15 cm, V = 15 kV, cone dimensionl base 3 cm, height 8 cm), (**c**) Mapping of electric field lines, and (**d**) Variation of electric field magnitude along z-axis, which presents the distance between the feeder and collector.

COMSOLMULTIPHYSICS4.2 software (Burlington, MA, USA) has been used to analyze the electric field distribution of the proposed electrode spinneret. Both practical dimensions and material properties are used for the Finite Element Method (FEM) calculations, which enable the visualization of the electric field intensity profile with understanding how this profile may be influenced by the spinneret geometry as well as material characteristics. The electrostatic interface is set to stationary, since the values of the field do not change over time. The physical geometries of the electrospinning spinnerets are established according to their practical dimensions, as shown in Figure 3 and Figure 4. In the presented simulations, the electrospinning setup is surrounded by a simulation sphere in which the upper part is assumed zero charge, while the lower part is grounded. A high potential is applied to the spinneret, while the collector and boundaries are zero potential. A mesh is created by the software, and finally the electric field intensity profile is obtained.

## 4. Results and Discussion

### 4.1. Electric Field Distribution

Both Figure 4a,b show the designed conical feeder in COMSOL software with electric field distribution analysis around the cone. The field is compact and concentrated around the cone, with field peaks near the cone edge due to the higher surface charge density. Figure 4c shows the mapping of electric field lines around the conical emitter. Figure 4d shows the decay of electric field magnitude along with the distance from the electrode down to the collector. The electric field strength is estimated a few millimeters from the nozzle to avoid peaks near the edges. Also, the convergent value of E is determined for comparison between the cone and needle electrodes. At the same applied voltage, the cone electrode shows strong field near the nozzle 3.226 × 10^5^ V/m. Also, it has higher convergent value of electric field near the collector 3.549 × 10^4^ V/m. For the needle case with is shown in Figure 3, the field near the nozzle recorded 1.834 × 10^5^ V/m, while the convergent value is 0.9 × 10^4^ V/m, as clarified in Figure 3d. A comparison between the values of E between cone and needle electrode is shown in Table 2.

At the same applied voltage, the electric field intensity in case of cone electrode is much stronger than the needle electrode. This leads to efficient power consumption. The strong field around the nozzle makes the cone electrode ideal to extract the jet and initiate electrospinning process in viscous solutions. Stronger field results in a fast initiation of fiber formation, increases the acceleration of the jet towards the collector, which leads to decreasing flight time and fast deposition of fibers on collector. The distance traveled should be sufficient to allow time for the solvent evaporation and solidification of the resulting fibers, which is about 15 cm, is found to be optimum.

### 4.2. Comparison between Typical Setup and Proposed Setup

The results of electric field analysis are extracted to be used in the simulation of dynamical behavior of electrospinning jet and then to make a comparison of the jet path in case of the simulated setup and practical one. Gradient of Electric field along the electrospinning axis (z-axis) is fitted into an equation, using curve fitting tools. The obtained equation is tested and compared to the values of electric field distribution from the Finite Element Analysis.

The obtained expression describes the electric field distribution along the electrospinning axis. The expression is used to estimate the value of electric field anywhere along the axes of electrospinning. This expression replaces the term V_0_/h in Equation (3) as previously mentioned. Matlab 9.1 software is used to simulate the jet path in both setup configurations using the modeling equations stated in the previous Section 3.1 with the modified form of Equation (3). The simulation parameters are given in Table 3.

**Figure 5:**
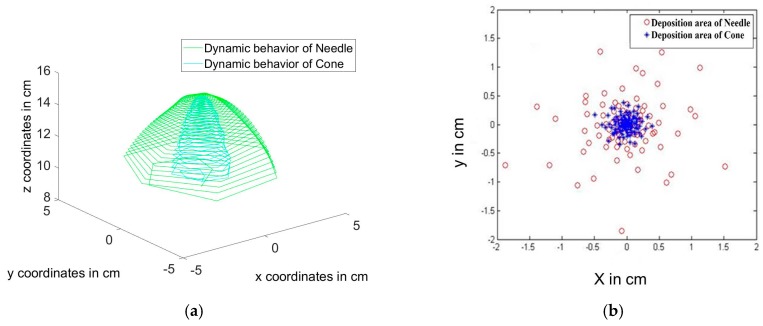
(**a**) Dynamic behavior analysis of the fibers jet of both cone and needle feeders and (**b**) Expected deposition area due to both feeders at specific z-value.

**Figure 6:**
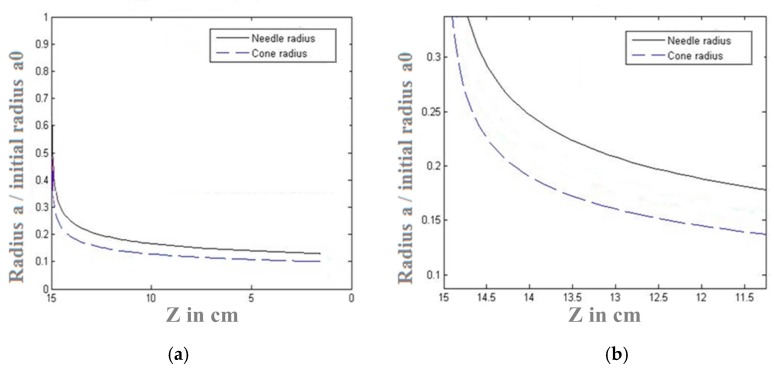
(**a**) Simulation results of radius of nanofibers relative change with distance due to both conical and needle feeders, with zoomed curves in (**b**).

Figure 5a shows the difference between the electrospinning jet path for both needle and conical feeders, the electrospinning jet in case of cone electrode is more compact. The cone feeder is found to have an influence on damping the effect of bending instability. Figure 5b shows the simulated deposition area of the electrospun jet generated from both needle and conical feeders. The conical feeder is theoretically expected to generate more dense nanofibers in a smaller deposited area when compared to the deposition area of the traditional needle feeder. Both Figure 6a,b refer to the reduction of nanofibers’ radius in conical feeder compared to the needle feeder along with the distance between both feeder and collector.

### 4.3. Experimental Results

**Figure 7:**
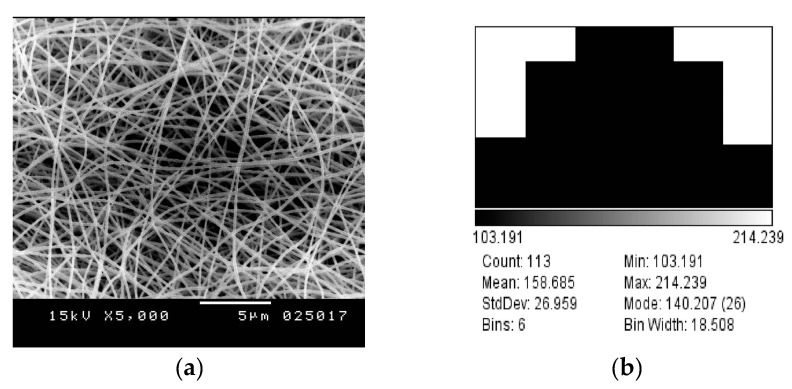
(**a**) Poly(vinyl alcohol) (PVA) 10% needle electrode at 15 cm, 1 mL/h. and 25 kV. (**b**) Mean diameter distribution of produced fibers by needle electrode.

**Figure 8:**
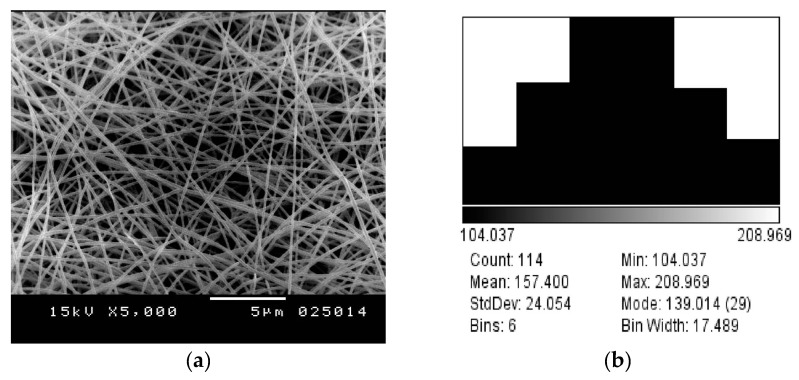
(**a**) PVA 10% cone electrode at 15 cm, 1 mL/h, and 25 kV. (**b**) Mean diameter distribution of produced fibers by cone electrode.

Experimentally, the produced nanofibers mats are further analyzed using SEM due to both conical and needle feeder at same voltage value of 25 KV at distance of z = 15 cm, as shown in both Figure 7 and Figure 8. The measured diameter in case of conical feeder is 157.4 ± 24.1 nm, when compared to diameter measurement of electrospun nanofibers generated from needle nanofiber to be 158.7 ± 27 nm. Therefore, that confirms the contribution of focused-field conical feeder that with larger feeding diameter of around 2 mm, the generated nanofibers have nearly same diameter as compared to the smaller traditional needle of input diameter around 1 mm.

**Figure 9:**
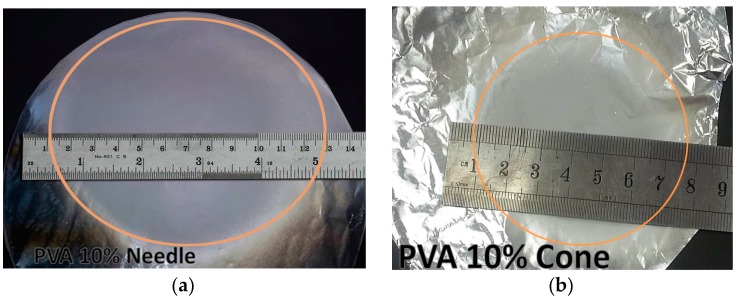
Deposition area of (**a**) Needle electrode (**b**) Cone electrode at same electrospinning process conditions (Material: PVA 10 wt %, Processing conditions: z = 15 cm, voltage = 25 kV, flow rate = 1 mL/h, Deposition time = 30 min).

Figure 9 shows the deposition area of nanofibers from both conical and needle feeders. It can be observed that the conical feeder generates concentrated mat with smaller area when compared to the needle feeder outcome, which is in consistence with the modeling results explained previously in Figure 5.

During electrospinning of fibers using the cone spinneret, it is noticed that the deposition area of fibers is greatly reduced. The conical feeder reduced deposition area of the fiber mat to a diameter ~6.8 cm, as shown in Figure 9, when compared to deposition area of diameter of ~12 cm in the case of needle electrode or covering most of the collector plate. In terms of deposition area, the new setup using cone spinneret has the ability to reduce deposition area and to restrict it to a small spot. It is expected that by increasing the dimensions of the cone electrode, they lead to an increase in the value of convergent electric field. While the maximum electric field decreases due to an increased size of the cone and a decrease in the sharp edges. Increasing the value of electric field will increase the directivity of the jet towards collector, decrease flight time, less number of bending cycles, more compact and limited deposition area, and increased fibers diameter [18]. There is a trade off between restricting deposition area and having finer nanofibers. Therefore, a further study is required to optimize the performance of the proposed electrode and compromise between the diameter of produced fibers and the desired deposition area.

**Figure 10:**
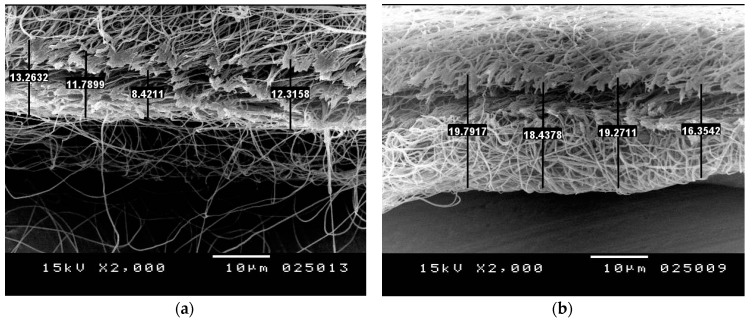
SEM image of cross section areas of nanofibers genrated from both (**a**) needle feeder and (**b**) conical feeder.

Some samples of cross section of the resulting fiber mats from both studied feeders are identified using SEM, as shown in Figure 10. It can be shown that using cone spinneret in electrospinning leads to a fiber mat with higher fiber density (a thicker denser layer).

Further analysis of SEM results from the two electrodes, the fiber packing density of resulting fiber mats can be roughly estimated, as shown in both Table 4 and Table 5. This can be achieved through calculation of the ratio of effective area of fibers to total area and taking into consideration the thickness of the resulting mat. Using Image j software, the effective area of fibers can be determined by measuring the area of gaps (i.e., porosity) and subtracting from total area. The thickness of the fiber mats resulting from needle and cone electrodes are 11.4475 and 18.4638, respectively, as shown in Figure 10. The ratio of effective area of fibers to total area is found to be ~0.9 in case of needle electrode and ~0.8676 for cone electrode. Therefore, the ratio of fiber packing density of cone electrode is ~1.5549 that of needle electrode. This indicates that the cone electrode has a higher production rate than the needle electrode.

## 5. Conclusions

This paper shows the impact of conical feeder inside electrospinning process on the generated nanofibers mat. The conical feeder shows more focused and higher intense electric field around it, which leads to more concentrated nanofibers with smaller deposition area and thicker mat when compared to the traditional needle electrode. When using the conical feeder, deposited fibers are highly concentrated to a smaller circular area of diameter ~6 cm, instead of a doubled deposition area in the case of a traditional needle spinneret. This shows that the cone electrode can be used to dampen the bending instability and reduce its effect. It can restrict the deposition of nanofibers to small areas, which is suitable for many applications that require accurate deposition of nanofibers. It can be quite important to applications like solar cells or sensors. Moreover, the proposed setup manages to generate electrospun nanofibers at lower voltages as compared to typical electrospinning setup with no beads formed, which leads to efficient power consumption.

## Figures and Tables

**Table 1:** Dimensionless parameters and groups [7,16].

Symbols	Parameters	Equation
L	Length scale	$L={\left(\frac{e^{2}}{{{\pi a}_{0}}^{2}G}\right)}^{\frac{1}{2}}$
Q	charge	$Q=\frac{e^{2}\mu^{2}}{L^{3}{\mathrm{mG}}^{2}}$
V	Voltage	$V=\frac{{\mathrm{eV}}_{0}\mu^{2}}{{\mathrm{hLmG}}^{2}}$
$F_{\mathrm{ve}}$	Elastic modulus	$F_{\mathrm{ve}}=\frac{{{\pi a}_{0}}^{2}\mu^{2}}{\mathrm{mLG}}$
A	Surface tension	$A=\frac{{{\alpha \pi a}_{0}}^{2}\mu^{2}}{{\mathrm{mL}}^{2}G^{2}}$
H	Distance from pendant drop to collector	$H=\frac{h}{L}$
$\bar{t}$	Time	t¯=tμG
$\bar{l}$	Length of rectilinear part of jet	$\bar{l}=\frac{1}{L}$
$\bar{\vartheta }$	Velocity	ϑ¯=ϑLGμ
$\bar{\sigma }$	Stress	$\bar{\sigma }=\frac{\sigma }{G}$

**Table 2:** Comparison of the values of E between cone electrode and the typical electrospinning setup.

Electrode	Maximum E near nozzle (few millimeters from nozzle) in V/m	Convergent E in V/m
Needle	1.834 × 10^5^	0.9 × 10^4^
Cone	3.226 × 10^5^	3.549 × 10^4^

**Table 3:** Discrete bead model simulation Parameters [7,16].

Symbol	Definition	Values in SI units	Values in gaussian units
a_0_	Initial jet radius	150 µm	150 × 10^−4^ cm
h	Distance from pendant drop to collector	0.15 m	15 cm
$V_{0}$	Applied voltage	10 kV	10,000/299.8 statV
α	Surface tension	0.7 k gs^−3^	700 gs^−3^
µ	Viscosity	10^3^ kg/(m·s)	1 × 10^4^ g/(cm·s)
G	Elastic modulus	10^5^ kg/(m·s^2^	10^6^ g/(cm·s^2^
e	Charge of bead	2.83 × 10^−4^ C	8.48 statC
m	Mass of bead	0.293 × 10^−8^ kg	0.283 × 10^5^ g
w	Frequency of perturbation	10^4^·s^−1^	10^4^·s^−1^
λ	Wavelength of perturbation	10^−4^ m	10^−2^ cm

**Table 4:** Calculations of fibers’ effective area.

Electrode	Area of gaps ${\left(\mu m\right)}^{2}$			Average area of gaps ${\left(\mu m\right)}^{2}$	Ratio of effective area to total area
	Region 1 40 ${\left(\mu m\right)}^{2}$	Region 2 40 ${\left(\mu m\right)}^{2}$	Region 3 40 ${\left(\mu m\right)}^{2}$		
Needle	4.872	3.158	3.348	5.29	0.867
Cone	3.659	6.882	5.342	3.795	0.900

**Table 5:** Summary of performance for the two studied spinnerets.

Comparison aspect	Needle	Cone
Fibers diameters	~158 ± 27 nm	~157 ± 24 nm
Beads formation	No beads	No beads
Deposition area	Large diameter > 12 cm covering most of the collector	compact Reduced Diameter ~ 6.8 cm
Fiber density	Low density	dense
